# Factors Associated with Breast Cancer Screening Adherence among Church-Going African American Women

**DOI:** 10.3390/ijerph18168494

**Published:** 2021-08-11

**Authors:** Pooja Agrawal, Tzuan A. Chen, Lorna H. McNeill, Chiara Acquati, Shahnjayla K. Connors, Vijay Nitturi, Angelica S. Robinson, Isabel Martinez Leal, Lorraine R. Reitzel

**Affiliations:** 1 Department of Psychological, Health and Learning Sciences, University of Houston, Houston, TX 77204, USA; poagrawa@utmb.edu (P.A.); tchen3@central.uh.edu (T.A.C.); connorss@uhd.edu (S.K.C.); vnitturi@central.uh.edu (V.N.); imarti31@central.uh.edu (I.M.L.); 2 School of Medicine, University of Texas Medical Branch, Galveston, TX 77555, USA; 3 Health Research Institute, University of Houston, Houston, TX 77204, USA; 4 MD Anderson Cancer Center, Department of Health Disparities Research, Houston, TX 77230, USA; lmcneill@mdanderson.org (L.H.M.); cacquati@central.uh.edu (C.A.); 5 Graduate College of Social Work, University of Houston, Houston, TX 77204, USA; 6 Department of Social Sciences, University of Houston-Downtown, Houston, TX 77002, USA; 7 Department of Radiology, University of Texas Medical Branch, Galveston, TX 77555, USA; an3robin@utmb.edu

**Keywords:** breast cancer screening, mammogram, cancer health equity, African American women, cancer survivorship, Andersen behavioral model, predisposing factors, enabling factors, need factors

## Abstract

Relative to White women, African American/Black women are at an increased risk of breast cancer mortality. Early detection of breast cancer through mammography screening can mitigate mortality risks; however, screening rates are not ideal. Consequently, there is a need to better understand factors associated with adherence to breast cancer screening guidelines to inform interventions to increase mammography use, particularly for groups at elevated mortality risk. This study used the Andersen Behavioral Model of Health Services Use to examine factors associated with adherence to National Comprehensive Cancer Network breast cancer screening guidelines amongst 919 African American, church-going women from Houston, Texas. Logistic regression analyses measured associations between breast cancer screening adherence over the preceding 12 months (adherent or non-adherent) and predisposing (i.e., age, education, and partner status), enabling (i.e., health insurance status, annual household income, employment status, patient-provider communication, and social support), and need (i.e., personal diagnosis of cancer, family history of cancer, and risk perception) factors, separately and conjointly. Older age (predisposing: OR = 1.015 (1.007–1.023)), having health insurance and ideal patient–provider communication (enabling: OR = 2.388 (1.597–3.570) and OR = 1.485 (1.080–2.041)), and having a personal diagnosis of cancer (need: OR = 2.244 (1.058–4.758)) were each associated with greater odds of screening adherence. Only having health insurance and ideal patient-provider communication remained significantly associated with screening adherence in a conjoint model; cancer survivorship did not moderate associations between predisposing/enabling factors and screening adherence. Overall, results suggest that interventions which are designed to improve mammography screening rates amongst African American women might focus on broadening health insurance coverage and working to improve patient–provider communication. Implications for multi-level intervention approaches, including the role of churches in their dissemination, are proposed.

## 1. Introduction

Breast cancer is the most commonly diagnosed cancer among women in the United States, except for skin cancers [1]. The average risk of a woman developing breast cancer in her lifetime is approximately 13% and the American Cancer Society (ACS) estimates that 281,550 new cases of invasive breast cancer and 49,290 cases of ductal carcinoma in situ (DCIS) will be diagnosed in 2021 [1]. Breast cancer is also the second leading cause of cancer death in women after lung cancer [1]. It is estimated that 43,600 women will die from breast cancer in 2021 [1].

African American/Black (hereafter referred to as African American) women have an unequal burden of breast cancer mortality. The overall breast cancer incidence rate in African American women is 126.5 cases per 100,000 compared to 130.1 in White women [2]. Although the incidence rate is lower among African American women, breast cancer mortality is approximately 40% higher compared with White women [2]. African American women are also twice as likely as women of other racial and ethnic backgrounds to be diagnosed with triple-negative breast cancer [2,3] and tend to receive a diagnosis of breast cancer at more advanced stages [4].

Although African American women present with more aggressive cancers as compared to White women, disparities in mortality remain after accounting for tumor stage and characteristics. Thus, the racial disparity may be partially attributed to other factors, such as rates of screening mammograms [5]. Prior to 2015, African American women had lower rates of mammography screening as compared to White women [6]. In more recent years, the rates of mammography screening have increased among African American women, but nevertheless remain below the 81% Healthy People 2020 objective [6,7]. 

There are several working recommendations for who should be screened for breast cancer, at what age, and at what frequency, and these recommendations may differ based on the associated professional groups espousing them. A relatively clear recommendation is that of the National Comprehensive Cancer Network (NCCN), which recommends annual screening mammography beginning at age 40 years for all women [8]. These recommendations are also consistent with those of the American College of Radiology and the Society of Breast Imaging [9]. Routine breast cancer screening, such as that promoted by the NCCN, can reduce mortality and improve survival outcomes [10]. 

Given the disproportionally high rate of mortality among African American women with breast cancer, it is important to examine factors that are associated with breast cancer screening adherence in order to better understand factors affecting adherence to recommended screening guidelines in this population. The Andersen Behavioral Model of Health Services Use can be used to examine factors that lead to the use of health services [11,12,13], including obtaining cancer screenings as recommended [14,15]. According to this model, health service utilization depends on three separate categories: predisposing, enabling, and need factors [11,12,13]. Predisposing factors are existing conditions/characteristics that influence people to use or not use services (e.g., age, partner status, education, race/ethnicity, religiosity and fatalism) [11,12,13]. Enabling factors are those that facilitate or impede the use of services, including financial means to access services (e.g., income and insurance status) [11,12,13]. Need factors are those that laypeople or health care providers recognize as requiring medical treatment and can be divided into perceived and evaluated need (e.g., diagnosis of a medical condition, such as cancer, or family history) [11,12,13].

The Andersen model is helpful to compare whether one category of factors has a greater effect on breast cancer screening rates over another, an aim that has not been explored previously among African American women. For example, enabling factors may have the largest influence on screening for African American women, who are more likely to encounter various societal and systemic barriers that lead to increased risk for being uninsured and low-income relative to White women [16,17,18]. Even if breast cancer screenings are available free of charge, low-income individuals may face barriers to receiving them (e.g., no childcare to allow a health provider visit, difficulty with obtaining transportation) [19]. On the other hand, need factors may drive breast cancer screening adherence given the burden of breast cancer mortality experienced by this group, or they may moderate the association between enabling and/or predisposing factors and screening adherence. 

Few previous studies have employed a theoretical framework, such as the Andersen model, as a guide to selecting variables that may be associated with breast cancer screening adherence [14]. One notable recent study that used the Andersen model to examine factors associated with breast cancer screening among Indigenous women found that, among predisposing factors, age was a significant predictor of breast cancer screening [14]. Among enabling factors, women with more formal education were more likely to undergo breast cancer screening [19]. Among need factors, participants with family cancer history were more likely to undergo breast cancer screening [19]. The extent to which this would generalize to African American women is not known; however, similar to Indigenous women, some African American women have lower median wages and employment rates than White women. Thus, these factors are also critically important to examine in this racial group [20]. 

With regard to studies on factors that influence the use of mammography screening among African American women specifically, one important work is Orji and colleagues’ literature review on this topic [19]. This literature review identified 24 articles published between 2005 and 2017; however, only 45.9% used a theory to guide variable selection and analysis [19]. Notably, the literature review itself used the Andersen model to synthesize prior work [19]. They reported that among predisposing factors, educational achievement, basic knowledge of breast cancer, and recommended screening frequency were positively associated with mammography screening, with 25% of the studies finding a positive association between education and screening adherence [19]. Medical mistrust was negatively associated with mammography use. Age, breast cancer beliefs (e.g., family teachings, the idea that surgery could cause cancer to spread), religiosity and fear and fatalism had mixed results, indicating a need for further research [19]. Among enabling factors, 10 (42%) of the studies identified health insurance as positively associated with mammography intentions or screening history [19]. Accessibility of care, income, and healthcare utilization (e.g., receiving regular medical checkups or physician visits within the last year) were other enabling factors found to be positively associated with mammography screening. Among need factors, physician recommendation and personal/family history of breast cancer were positively associated with mammography screening, while pain and discomfort caused by mammography and familial responsibilities (e.g., family duties or caretaking demands) were negatively associated with mammography screening [19]. Although Orji’s and colleagues’ work was a significant step forward in summarizing factors associated with breast cancer screening adherence among African American women across extant studies, it is notable that many of these studies did not comprehensively examine predisposing, enabling, and need factors and most of the included studies (58.4%) had relatively small sample sizes (i.e., less than 300 women) [19].

The current study builds upon the extant literature regarding African American women and factors associated with breast cancer screening using the Andersen framework while adding to the literature by examining additional variables that were not explored previously, such as the role of the patient–provider relationship (i.e., how often the patient felt listened to or respected by the physician), a potential enabling variable of relevance [15]. Specifically, the purpose of the current study was to: (1) Examine associations between predisposing, enabling, and need factor variables and with breast cancer screening adherence; (2) Explore whether predisposing, enabling, and/or need factor categories have the strongest association with breast cancer screening adherence; and (3) Investigate the potential moderating effect of need factors on the associations between predisposing factors and enabling factors, respectively, and breast cancer screening adherence among a large convenience sample of >900 church-going African American women. While religiosity and fatalism have been measured in prior studies among African American women with mixed results, this study is one of only a few studies conducted among a church-based sample [21,22]. This is important because 47% of African Americans in the United States attend religious services at least once a week [23,24]. Furthermore, there may be less variability in constructs previously yielding mixed results, such as religiosity, in homogeneous church-based samples relative to more heterogeneous community-based samples. 

Better understanding of the associations between factors and breast cancer screening adherence among a church-based sample of African American women may have several practical applications. For example, it may help inform cancer-reducing interventions delivered within the church or church ministry, which can be tailored to reflect connections with relevant scripture. Moreover, churches may be an important means of dissemination of health information, such as cancer screening guidelines, which can be promoted to groups of women at elevated risk of screening non-adherence. Additionally, the identification of predisposing, enabling, and/or need factors essential to improving breast cancer screening among African American women may also help physicians provide increased support and health education to their patients in order to increase adherence to screening recommendations [14,21,25,26]. Further, the potential findings of this study may help inform policies to remove economic barriers that limit access to screening.

## 2. Materials and Methods

### 2.1. Design

Participants comprised a convenience sample from three churches in Southwest Houston, Texas, with predominantly African American membership that agreed to participate in data collection aimed at understanding health and cancer risk among African American adults. Recruitment occurred through printed and televised media in the churches and in-person solicitation. Participants were required to be ≥18 years old, living in the Houston area with a functional telephone number, and an attendee of one of the participating churches. Surveys were completed on-site using a computerized questionnaire format. As compensation, participants received a $30 gift card. 

A previous publication details the parent study design and the characteristics of the participating churches and their participants [27]. In short, the underlying parent study, Creating a Higher Understanding of cancer Research and Community Health (Project CHURCH), was a community-based participatory research project with an aim of understanding disparities in cancer risk factors and engaging African Americans as partners in the research process in order to reduce barriers to research participation and improve research outcomes. Project CHURCH used a longitudinal cohort design and started in 2008 with a large Houston mega-church, expanding to add two additional, smaller churches in 2012. Procedures were informed by the church and scientific advisory board. Overall, Project CHURCH enrolled ~1500 adults from the original church, and around 400 adults in each of the smaller churches. About 70% of participants were women, mirroring their proportion amongst church attendees [27]. This study was approved by the IRBs associated with the University of Texas MD Anderson Cancer Center (protocol code 2007-0970, approved 2/28/08; protocol code 2012-0051, approved 2/14/12) and the University of Houston (protocol code 14423-EX, approved 7/10/14).

The number of participants targeted for these data collections varied based on the size of the participating churches and totaled 2254 (*N* = 1666 women). For the present study, only women 40 years of age or older (*N* = 1108) and with complete data on all included measures (*N* = 919) comprised the final analytic sample.

### 2.2. Measures

#### 2.2.1. Predisposing Factors

Predisposing factors included age (in years), educational achievement (≤high school degree, some college, vs. ≥Bachelor’s degree), and partner status (married or living with partner vs. divorced, widowed, separated, and never married). Factors were included in the predisposing category based on precedent from previous research and guidance by the Andersen model [11,12,13,19].

#### 2.2.2. Enabling Factors

Enabling factors included whether the participant was covered by health insurance over the last year (no vs. yes), annual household income (<$40,000, $40–$79,999, vs. ≥$80,000) employment status (not employed vs. employed for wages/self-employed), patient–provider communication, and social support.

Patient–provider communication was included as an enabling factor due to a similar categorization in a prior study on prostate cancer screening [15]. Patient–provider communication was measured by four items from the Consumer Assessment of Healthcare Providers and Systems (CAHPS) survey. Participants were asked how often their health care provider or doctor over the prior year: (1) listened carefully to them, (2) explained things in a way they could understand, (3) showed respect for what they had to say, (4) spent enough time with them, with response choices of: never, sometimes, usually, and always. Cronbach’s alpha for these four items in this sample was 0.90. Based on convention [28], the measure was scored based on whether each participant selected “always” to each item vs. did not select “always” for each item, essentially capturing ideal versus not always ideal patient–provider communication. 

Social support was included as an enabling factor as recommended by an updated version of the Andersen model [13]. Social support was measured through the Interpersonal Support Evaluation List (ISEL)-12, which consists of 12 items and 3 subscales [29]. The three subscales were the tangible support subscale (availability of material aid), the belonging subscale (availability of companions with whom one may engage in activities), and the appraisal subscale (availability of emotional support). Items were rated on a four-point scale (1 = definitely false to 4 = definitely true), with subscale scores ranging from 4 to 16. Higher scores indicated greater social support [30]. Cronbach’s alpha for ISEL-12 subscales in this sample were 0.63 (tangible support), 0.70 (belonging support), and 0.70 (appraisal support). 

#### 2.2.3. Need Factors

Need factors included a personal diagnosis of cancer (any type; no vs. yes), family history of cancer (any type; yes vs. no), and risk perception. Personal cancer history and family history of cancer were included in need factors based on similar categorization in a previous study, and because need factors include an individual’s perceived or evaluated need [14]. Risk perception was measured through three discrete items from the Health Information National Trends Survey (HINTS) [31]. The first item was: “How likely do you think it is that you will develop any type of cancer in the future? Would you say your chance of getting cancer is…” Answer options ranged from 1 = very low, 2 = somewhat low, 3 = moderate, 4 = somewhat high, to 5 = very high. The second item asked: “Compared to the average person your age, would you say that you are…” Answer options ranged from 1 = more likely to get cancer, 2 = less likely, to 3 = about as likely. The final item was: “How often do you worry about getting some type of cancer?” Answer options were: 1 = never, 2 = rarely, 3 = sometimes, 4 = often, and 5 = all the time. Each of the risk perception items were respectively included in the analysis. 

#### 2.2.4. Breast Cancer Screening Adherence

Receipt of breast cancer screening was measured by asking participants whether they had ever had a mammogram (0 = no; 1 = yes) and, for those endorsing “yes” to the lifetime mammography item, when their most recent mammogram had taken place. Those reporting having received a mammogram 1 year ago or less were considered adherent, whereas those indicating their last mammogram was >1 year ago were considered not adherent, in accordance with NCCN screening recommendations [7].

#### 2.2.5. General Participant Characteristics

Data were also collected about the number of family members under the age of 18 in the household and reasons for obtaining the most recent mammogram for descriptive purposes.

### 2.3. Data Analysis

The descriptive statistics of participants’ characteristics and study variables were assessed. The comparisons between those excluded due to missingness vs. analytic sample on participant characteristics were examined. Participant characteristics relative to breast cancer screening adherence were examined using t-test or chi-square test for continuous and categorical variables, respectively. 

For the main analyses (Aim 1), separate logistic regression analyses were conducted to measure associations between breast cancer screening adherence and predisposing factors (i.e., age, education, and partner status), enabling factors (i.e., health insurance status, annual household income, employment status, patient–provider communication, and ISEL social support subscales), and need factors (i.e., personal diagnosis of cancer, family history of cancer, and risk perception). Next (Aim 2), predisposing, enabling, and needs factors were all included in the logistic regression analysis predicting breast cancer screening adherence. Lastly (Aim 3), to examine the moderation effect of need factors, the interaction terms of the significant need factor and predisposing/enabling factor were included in the models. Separate moderation effects were examined. Continuous variables were mean-centered prior to moderation analyses, and church site (1, 2, or 3) was included as a covariate in all logistic regression analyses. All analyses used two-tailed tests of significance with a statistical significance level designated at *p* < 0.05. All analyses were conducted using SAS version 9.4 [32].

## 3. Results

Of the 1108 total female participants aged 40 and above in the parent study, 919 (82.94%) had full information on all the measures included in this study. Women 40 or older with missing data (*N* = 189) differed statistically from those who had all data (*N* = 919) on ISEL belonging subscale scores, recruitment site, health insurance status, and screening adherence. Specifically, those with missing data reported higher ISEL belonging subscale scores (13.65 vs. 13.22, *p* = 0.0304), were less likely to have health insurance (74.47% vs. 88.36%, *p* < 0.0001), were more likely to be recruited from church site 3 (29.10% vs. 19.15%, *p* = 0.0071) and were less likely to be screening adherent (56.38% vs. 67.03%, *p* = 0.0052) relative to participants who had full data on all the measures. 

### 3.1. Participant Characteristics

Participants ranged from age 40 to 86 (M = 53.12, SD = 8.56). Of the 919 participants, 67.03% (*N* = 616) were screening adherent. Overall, 11.43% of this group (*N* = 105) reported education ≤ high school, 40.70% some college (*N* = 374), and 47.88% ≥ bachelor’s degree (*N*= 440). Additionally, 42.66% (*N* = 392) reported they were married/living with a partner, 88.36% (*N* = 812) reported they were insured, 69.75% (*N* = 641) reported they were employed, 32.21% (*N* = 296) reported ideal patient-provider communication, 5.01% (*N* = 46) had been diagnosed with at least 1 cancer, and 59.74% (*N* = 549) reported a family history of cancer(s). Participant characteristics by breast cancer screening adherence status are shown in Table 1.

Compared with participants who were screening non-adherent, participants who were breast cancer adherent were older (54.09 vs. 51.14, *p* < 0.0001) and had higher ISEL tangible support scores (14.06 vs. 13.67, *p* = 0.0276). Chi-square tests revealed significant associations between screening adherence and church site (*p* = 0.0109), health insurance status (*p* < 0.0001), patient–provider communication (*p* = 0.0033), and personal diagnosis of cancer (*p* = 0.0472). Those reporting screening adherence were more likely to have been recruited from church site 3 (21.75% vs. 13.86%), have health insurance (92.53% vs. 79.87%), report ideal patient–provider communication (35.39% vs. 25.74%), and have been a cancer survivor (6.01% vs. 2.97%) relative to participants who were non-adherent with NCCN breast cancer screening recommendations.

### 3.2. Aim 1: Predisposing, Enabling, and Need Factors and Breast Cancer Screening Adherence

Logistic regression analyses revealed that among the predisposing variables, older participants had greater odds of breast cancer screening adherence (OR: 1.015, 95% CI: 1.007–1.023). Among the enabling factors, participants who were insured and who had ideal patient–provider communication had 2.388 (95% CI: 1.597–3.570) and 1.485 (95% CI: 1.080–2.041) times greater odds of breast cancer screening adherence, respectively. Finally, among the need factors, cancer survivors had 2.244 (95% CI: 1.058–4.758) greater odds of breast cancer screening adherence relative to those who had never been diagnosed with cancer(s) (Table 2).

### 3.3. Aim 2: Predisposing, Enabling, vs. Need Factors and Breast Cancer Screening Adherence

When all factors were simultaneously included in the model, logistic regression analysis showed that two enabling factors, health insurance and patient-provider communication, were significantly associated with screening adherence. Specifically, participants who had health insurance (OR: 2.286, 95% CI: 1.464–3.570) and who reported ideal patient–provider communication (OR: 1.528, 95% CI: 1.104–2.115) had greater odds of breast cancer screening adherence (Table 2). 

### 3.4. Aim 3: Moderation Effects of Personal Cancer Diagnosis (i.e., Survivorship)

The only significant need factor, personal diagnosis of cancer, was examined as a moderator in the association of the significant predisposing factors and enabling factors, respectively, with breast cancer screening adherence. To examine the moderation effect, the interaction terms of personal diagnosis of cancer and age/health insurance/patient-provider communication were included separately in the models. None of the interaction terms were significant, implying that a personal diagnosis of cancer did not moderate the relationship between age (*p* = 0.0679), health insurance (*p* = 0.695), or patient-provider communication (*p* = 0.425) and breast cancer screening adherence (data not shown).

## 4. Discussion

Among this sample of church-going African American women, 67% reported receiving a screening mammogram within the last year. This finding was slightly lower but comparable to the 68.1% of African American women in the United States who reported receiving a mammogram within the past 2 years (data from 2018) [33]. However, prevalence rates remain well below the Healthy People 2020 objective of 81%, indicating a considerable need for interventions that increase breast cancer screening [6,7]. Better understanding of factors associated with breast cancer screening adherence amongst African American women, who are at relatively higher risk for breast cancer mortality compared with White women [2], is important to inform intervention development and delivery. The present study, conducted solely among church-going African American women from three churches in Houston, Texas, investigated health care utilization factors that could help inform these future interventions to increase breast cancer screening rates. 

Among predisposing factors, increased age was associated with greater odds of receiving a screening mammogram within the last year. Some previous studies have also found that increased age is associated with screening adherence among African American women [34,35]. Decreased adherence to screening among younger women may be due to disputed screening guidelines about the age at which screening should be initiated (e.g., the U.S. Preventive Services Task Force (USPSTF) recommends biennial screening mammography for women aged 50 to 74 years [36].) Previous studies conducted among African American women have also found that older women tend to have a greater number of visits to their physician and often obtain mammograms as a result of physician recommendations [37]. Other studies among African American women, however, have found that older age is associated with a lower motivation to screen and the receipt of fewer mammograms [38,39]. Additional research is needed to understand why these inconsistencies exist and to provide tailored interventions to both older and younger African American women to increase their screening adherence.

Among enabling factors, participants who were insured and who reported ideal levels of patient–provider communication were more likely to be breast cancer screening adherent. Numerous studies have found insurance status to be associated with receipt of breast cancer screening [19]. Orji and colleagues’ literature review found that health insurance was the most frequently identified influence on mammography among African American women, with 42% (*N* = 10) of the included studies finding a significant association between health insurance and mammography intentions or adherence [19]. This is not surprising given that cost has been cited as a barrier to breast cancer screening among African American women [40,41]. Given that at least 8% of African American women in the United States were uninsured in 2019, future interventions designed to increase mammography rates may consider targeting uninsured women for free mammograms through mobile mammography units, community partnerships, or national programs [18,42,43,44]. Women who had ideal patient–provider communication also had greater odds of screening adherence. This indicates that a strong relationship between the patient and physician may influence a woman’s likeliness to obtain a mammogram, a finding seen in a previous study among African American women, but which has never been tested in the context of the Andersen model [45]. Consequently, future interventions to increase screening adherence may include the training of physicians to relay information in a manner that builds trust and respect through patient-centered communication [46,47]. Building trust and respect in the context of a clinical encounter, particularly when patient and provider race/ethnicity is mismatched, may be a time-intensive and ongoing endeavor; healthcare facilities employing physicians should promote ways in which spending this “extra time” can be incentivized (or at least not actively disincentivized). Likewise, women who do not report ideal patient–provider communication might be advised to seek another physician with whom more ideal communication might be possible. Alternatively, women may benefit from coaching regarding how they can be empowered to obtain clearer information from physicians in the context of medical visits in order to become more informed about their options and eligibility for breast cancer screening. This is especially important as most women in this sample reported receiving their last mammogram in the context of a routine physical exam (84.98%). It also bears mentioning that attention to “next steps” in the process is also critical for saving lives, which includes ensuring access to free diagnostic exams and breast biopsies in the event of a screening abnormality.

When need factors were assessed, those reporting a personal diagnosis of cancer were more likely to be screening adherent. Similar results have been reported in previous studies involving African American women, finding that women who reported personal exposure to breast cancer (through own diagnosis or knowing someone with breast cancer) were more likely to have had a mammogram in the past than women without personal exposure [48,49]. Additionally, previous evidence has supported that African American women with a history of chronic illnesses such as cancers were more regularly screened [49]. These findings indicate that personal diagnoses of cancer may contribute to increased knowledge that could potentially elevate the importance of regular screening. It should be noted that our cancer survivorship variable encompassed all cancer types, similar to at least one prior work [14], but in contrast to another contribution where breast cancer survivorship was specifically included [48]. However, having received a prior diagnosis of any cancer is a risk factor for the development of other/recurrent cancers [50], and thus may affect cancer screening adherence in general. Moreover, most survivors in this sample were breast cancer survivors (31/46 = 67.39%); thus, only 1.63% (15/919) of the sample were survivors of other cancers. Future studies with a larger group of survivors might consider an examination of breast cancer survivorship versus other cancer survivorship as relevant need factors.

Overall, the current work is the first study to examine factors associated with breast cancer screening using the Andersen model pursuant to Orji and colleagues’ recent literature review [19]. Although each of the above described predisposing, enabling, and need variables contributed unique variance within their overarching grouping to the prediction of breast cancer screening adherence, health insurance and patient-provider communication were the two variables that remained significant in the context of all others. These results suggest two potentially changeable factors that may increase adherence to screening—irrespective of a personal history of cancer—that might be prioritized in intervention design. A multi-level ecological intervention with the ability to: (1) address national and local policies and practices reducing un-insurance rates and/or targeting uninsured women for free cancer screenings; (2) train medical providers on culturally-appropriate and respectful ways to interact with patients; and (3) empower patients to seek and develop ideal health communication with their physicians, may contribute to improving breast cancer screening rates amongst African American women and reduce the gap between the status quo and Healthy People 2020 objectives [9,10].

Given the importance of churches in African American communities, these settings may be useful as a method of dispersing information about mammography screening to women with the goal of increasing screening awareness and adherence [24]. Previous research among this group has found that church-based breast cancer screening education programs positively affected mammography attainment [51]. Darnell and colleagues’ study found that African Americans who reported receiving information about mammograms numerous times at their churches were more likely to report mammography use within the prior year than those who encountered such information less frequently [52]. Future interventions may consider taking advantage of the reach and influence of churches to promote mammography screening among African American women, potentially with a particular focus on the development of health literacy and self-advocacy in the context of patient–provider interactions [53].

A limitation of this study is its cross-sectional nature as temporal relations cannot be determined. Other aspects of limitation include the use of self-reported measures for receiving screening mammograms, the timeframe of which may be affected by recollection bias. Additional factors not accounted for or assessed in this study (e.g., psychosocial factors, social network characteristics, mental health symptoms or diagnoses) may have affected the variables in this study, however, we did not examine these factors as they have not been traditionally included in the Andersen model. Statistical analysis was limited to variables collected in all three churches; therefore, some of the variables that have been previously included in the Andersen model could not be analyzed (e.g., health care utilization). Furthermore, this study used convenience sampling to select participants and was limited to church-going African American women in the South, which may affect generalizability to all African American church-going women, or to those who attend churches with lower proportions of African American membership. Moreover, this study did not collect information on which breast cancer screening guidelines were promoted within the respective participant treatment settings and/or followed by participants; thus, it is possible some women were following different screening guidelines than those promoted by the NCCN with which they were indeed adherent. Additionally, this study was largely a replication study, wherein factors examined in other studies were primarily included with the exploration of only a limited number of novel variables (e.g., patient–provider communication). Additional novel constructs may be important to examine in future work, and qualitative feedback from church attendees may be important in ascertaining factors of relevance. Finally, the present work used two-tailed tests based on prior mixed results in other studies, the direct (and novel) application of the Andersen framework in an African American sample, and the addition of limited new constructs; future confirmatory work would benefit from directional hypotheses, the use of one-tailed tests, and considerations for multiple testing.

Limitations are balanced by study strengths, including a large sample of church-going African American women from multiple recruitment sites, the use of a theoretical model guiding variable selection, and inclusion of factors such as patient-provider communication, which have not been extensively examined in the context of breast cancer screening among African American women. Future studies may consider using a longitudinal study design and including these additional variables.

## 5. Conclusions

In conclusion, this study expands upon existing literature by supporting the association between older age, health insurance coverage, ideal patient–provider communication, and cancer survivorship with adherence to mammography screening in a large sample of church-going African American women. Results have implications for intervention targeting and design. Specifically, (1) educational campaigns to reinforce the importance of establishing yearly mammogram screening behaviors might be best directed toward younger African American church-going women; such efforts might be powerful if congregant cancer survivors are part of educational campaigns; (2) policies and practices that reach and expand preventive (and diagnostic/curative) breast healthcare to uninsured African American women are needed; and (3) methods to enhance patient–provider communication may be important to increasing adherence to mammogram screening guidelines for those reporting less than ideal interactions with healthcare providers. Future interventions aimed at increasing mammogram screening rates among African American women should consider the potential role of the church as an ideal and culturally-relevant setting to provide information and connection to preventative care to their congregants. 

## Figures and Tables

**Table 1:** Participant Characteristics Relative to NCCN Breast Cancer Screening Adherence Status (*N* = 919).

	All	Non-Adherent	Adherent	*p*-Value
	919	303	616	
	Mean (SD)/% [n]			
Age	53.12 (8.56)	51.14 (8.29)	54.09 (8.53)	<0.0001
Education				0.0961
≤High school	11.43 [105]	11.88 [36]	11.20 [69]	
Some college	40.70 [374]	45.21 [137]	38.47 [237]	
≥Bachelor’s degree	47.88 [440]	42.90 [130]	50.32 [310]	
Partner Status				0.1461
Not married/living with a partner	57.34 [527]	60.73 [184]	55.68 [343]	
Married/living with a partner	42.66 [392]	39.27 [119]	44.32 [273]	
Number of family members <18 years old in house	0.48 (0.86)	0.54 (0.94)	0.45 (0.82)	0.1270
Church Site				0.0109
Site 1	67.36 [619]	70.30 [213]	65.91 [406]	
Site 2	13.49 [124]	15.84 [48]	12.34 [76]	
Site 3	19.15 [176]	13.86 [42]	21.75 [134]	
Health Insurance Coverage				<0.0001
No	11.64 [107]	20.13 [61]	7.47 [46]	
Yes	88.36 [812]	79.87 [242]	92.53 [570]	
Annual Household Income				0.0853
<$40,000	28.18 [259]	31.68 [96]	26.46 [163]	
$40,000–$79,999	38.19 [351]	39.27 [119]	37.66 [232]	
≥$80,000	33.62 [309]	29.04 [88]	35.88 [221]	
Employment Status				0.3874
Unemployed	30.25 [278]	28.38 [86]	31.17 [192]	
Employed	69.75 [641]	71.62 [217]	68.83 [424]	
Patient-Provider Communication				0.0033
Not always ideal	67.79 [623]	74.26 [225]	64.61 [398]	
Ideal	32.21 [296]	25.74 [78]	35.39 [218]	
Social Support				
ISEL Tangible	13.93 (2.42)	13.67 (2.58)	14.06 (2.32)	0.0276
ISEL Belonging	13.22 (2.60)	13.06 (2.82)	13.30 (2.49)	0.2191
ISEL Appraisal	14.02 (2.55)	13.80 (2.79)	14.12 (2.42)	0.0853
Personal Diagnosis of Cancer				0.0472
No	94.99 [873]	97.03 [294]	93.99 [579]	
Yes	5.01 [46]	2.97 [9]	6.01 [37]	
Family Diagnosis of Cancer				0.6668
No	40.26 [370]	41.25 [125]	39.77 [245]	
Yes	59.74 [549]	58.75 [178]	60.23 [371]	
Chance of Getting Cancer				0.8358
Very low	37.65 [346]	38.61 [117]	37.18 [229]	
Somewhat low	24.59 [226]	25.74 [78]	24.03 [148]	
Moderate	28.84 [265]	26.73 [81]	29.87 [184]	
Somewhat high	7.62 [70]	7.92 [24]	7.47 [46]	
Very high	1.31 [12]	0.99 [3]	1.46 [9]	
Likelihood of getting cancer compared to average person your age				0.8131
More likely to get cancer	7.83 [72]	8.58 [26]	7.47 [46]	
Less likely	57.13 [525]	56.11 [170]	57.63 [355]	
About as likely	35.04 [322]	35.31 [107]	34.90 [215]	
How often worry about getting cancer				0.660
Never	26.22 [241]	29.37 [89]	24.68 [152]	
Rarely	35.04 [322]	33.99 [103]	35.55 [219]	
Sometimes	31.56 [290]	30.03 [91]	32.31 [199]	
Often	5.11 [47]	4.62 [14]	5.36 [33]	
All the time	2.07 [19]	1.98 [6]	2.11 [13]	
Main reason for mammogram ^1^				<0.0001
Part of the routine physical exam	84.98 [781]	71.95 [218]	91.4 [563]	
Last mammogram was not normal	4.24 [39]	5.28 [16]	3.73 [23]	
A specific problem	3.81 [35]	3.96 [12]	3.73 [23]	
Something you heard/saw/read	0.33 [3]	0.66 [2]	0.16 [1]	
Family history	1.09 [10]	1.32 [4]	0.97 [6]	
You never had one and thought you should	0.44 [4]	1.32 [4]	0 [0]	

^1^ 47 women (5.11% of sample) who were non-adherent with NCCN breast cancer screening guidelines did not provide responses for the main reason for their most recent mammogram. ISEL = Interpersonal Support Evaluation List. SD = Standard deviation.

**Table 2:** Logistic Regression Analyses—Predicting Breast Cancer Screening Adherence (*N* = 919).

Factor	Effect	Estimate	SE	OR	95% CI	*p*-Value
Predisposing Factor	Church site 2	−0.213	0.205	0.808	(0.540, 1.208)	0.299
	Church site 3 (ref: Church site 1)	0.393	0.201	1.481	(0.998, 2.197)	0.051
	Age	0.015	0.004	1.015	(1.007, 1.023)	<0.0001
	Education (≥Bachelor’s degree) (ref: ≤High School)	−0.017	0.215	0.983	(0.644, 1.499)	0.936
	Education (Some college) (ref: ≤High School)	−0.317	0.214	0.729	(0.479, 1.108)	0.138
	Partner status (Married/Living with a partner) (ref: Other ^1^)	0.145	0.144	1.156	(0.873, 1.532)	0.311
Enabling Factor	Church site 2	−0.188	0.214	0.828	(0.545, 1.259)	0.378
	Church site 3 (ref: Church site 1)	0.433	0.202	1.541	(1.038, 2.288)	0.032
	Health insurance coverage (ref: No)	0.870	0.205	2.388	(1.597, 3.570)	<0.0001
	Annual household income ($40,000–$79,999) (ref: <$40,000)	0.064	0.188	1.066	(0.737, 1.542)	0.734
	Annual household income (≥$80,000) (ref: <$40,000)	0.242	0.204	1.274	(0.855, 1.898)	0.235
	Employment status (ref: Unemployed)	−0.256	0.167	0.774	(0.558, 1.075)	0.126
	Patient-provider communication (ref: Not Always Ideal)	0.395	0.162	1.485	(1.080, 2.041)	0.015
	ISEL Tangible support	0.009	0.039	1.009	(0.936, 1.089)	0.807
	ISEL Belonging	−0.032	0.036	0.969	(0.902, 1.040)	0.385
	ISEL Appraisal	0.012	0.037	1.012	(0.941, 1.088)	0.756
Need Factor	Church site 2	−0.074	0.222	0.929	(0.601, 1.435)	0.740
	Church site 3 (ref: Church site 1)	0.656	0.218	1.927	(1.258, 2.953)	0.003
	Personal diagnosis of cancer (ref: No)	0.808	0.384	2.244	(1.058, 4.758)	0.035
	Family members diagnosed with cancer (ref: No)	0.228	0.164	1.256	(0.911, 1.730)	0.164
	Chance of getting cancer (Somewhat low) (ref: Very low)	0.093	0.199	1.097	(0.744, 1.619)	0.641
	Chance of getting cancer (Moderate) (ref: Very low)	−0.083	0.311	0.921	(0.501, 1.693)	0.790
	Chance of getting cancer (Somewhat high) (ref: Very low)	−0.099	0.189	0.905	(0.625, 1.312)	0.600
	Chance of getting cancer (Very high) (ref: Very low)	0.436	0.732	1.547	(0.369, 6.492)	0.551
	Likelihood of getting cancer compared to average person your age (About as likely) (ref: More likely to get cancer)	0.208	0.209	1.232	(0.817, 1.856)	0.319
	Likelihood of getting cancer compared to average person your age (Less likely) (ref: More likely to get cancer)	0.397	0.171	1.487	(1.064, 2.078)	0.020
	How often worry about getting cancer (All the time) (ref: Never)	0.199	0.552	1.220	(0.413, 3.602)	0.719
	How often worry about getting cancer (Often) (ref: Never)	0.353	0.367	1.423	(0.693, 2.922)	0.337
	How often worry about getting cancer (Rarely) (ref: Never)	0.158	0.180	1.171	(0.822, 1.666)	0.382
	How often worry about getting cancer (Sometimes) (ref: Never)	0.205	0.199	1.228	(0.832, 1.812)	0.301
All	Church site 2	−0.128	0.237	0.880	(0.553, 1.401)	0.590
	Church site 3 (ref: Church site 1)	0.515	0.237	1.673	(1.052, 2.661)	0.030
	Age	0.004	0.008	1.004	(0.989, 1.020)	0.579
	Education (≥Bachelor’s degree) (ref: ≤High School)	−0.018	0.266	0.982	(0.583, 1.655)	0.946
	Education (Some college) (ref: ≤High School)	−0.289	0.248	0.749	(0.461, 1.217)	0.243
	Partner status (Married/Living with a partner) (ref: Other ^1^)	0.102	0.166	1.108	(0.801, 1.533)	0.537
	Health insurance coverage (ref: No)	0.827	0.227	2.286	(1.464, 3.570)	<0.0001
	Annual household income ($40,000–$79,999) (ref: <$40,000)	0.003	0.203	1.003	(0.673, 1.494)	0.989
	Annual household income (≥$80,000) (ref: <$40,000)	0.144	0.243	1.155	(0.717, 1.861)	0.553
	Employment status (ref: Unemployed)	−0.227	0.178	0.797	(0.562, 1.131)	0.204
	Patient-provider communication (ref: Not Always Ideal)	0.424	0.166	1.528	(1.104, 2.115)	0.011
	ISEL Tangible support	0.000	0.041	1.000	(0.922, 1.085)	0.992
	ISEL Belonging	−0.039	0.038	0.962	(0.893, 1.036)	0.303
	ISEL Appraisal	0.005	0.040	1.005	(0.930, 1.086)	0.900
	Personal diagnosis of cancer (ref: No)	0.711	0.392	2.036	(0.944, 4.392)	0.070
	Family members diagnosed with cancer (ref: No)	0.172	0.177	1.187	(0.839, 1.680)	0.334
	Chance of getting cancer (Somewhat low) (ref: Very low)	−0.008	0.211	0.992	(0.655, 1.500)	0.968
	Chance of getting cancer (Moderate) (ref: Very low)	−0.211	0.347	0.810	(0.410, 1.598)	0.543
	Chance of getting cancer (Somewhat high) (ref: Very low)	−0.140	0.198	0.869	(0.589, 1.282)	0.480
	Chance of getting cancer (Very high) (ref: Very low)	0.227	0.794	1.254	(0.264, 5.950)	0.776
	Likelihood of getting cancer compared to average person your age (About as likely) (ref: More likely to get cancer)	−0.118	0.319	0.888	(0.475, 1.660)	0.711
	Likelihood of getting cancer compared to average person your age (Less likely) (ref: More likely to get cancer)	0.056	0.332	1.058	(0.552, 2.028)	0.865
	How often worry about getting cancer (All the time) (ref: Never)	0.465	0.585	1.593	(0.506, 5.015)	0.427
	How often worry about getting cancer (Often) (ref: Never)	0.444	0.384	1.559	(0.734, 3.310)	0.248
	How often worry about getting cancer (Rarely) (ref: Never)	0.159	0.191	1.172	(0.807, 1.704)	0.405
	How often worry about getting cancer (Sometimes) (ref: Never)	0.202	0.210	1.224	(0.812, 1.847)	0.335

Note. SE = Standard Error; OR = Odds Ratio; CI = Confidence Interval; ^1^ Other included divorced, widowed, separated, and never married. ISEL = Interpersonal Support Evaluation List. SE = Standard Error. OR = Odds Ratio. CI = Confidence Interval. Ref = Reference group.

## Data Availability

The data are not publicly available due to ethical restrictions based on informed consent agreements that did not specify broad data release. Additionally, we worked with the churches’ leadership directly for permission to conduct the broader study, which banked sensitive data (e.g., buccal saliva sample); the broad release of de-identified data was not something that the churches agreed to for privacy/confidentiality reasons. Data may be available from the Ancillary Studies Committee (PI: McNeill) at the University of Texas MD Anderson Cancer Center for researchers who meet the criteria for access to confidential data. Interested researchers may contact Office of Human Subjects Protection at MD Anderson Cancer Center at IRB_Help@mdanderson.org or at 713-792-6477.
